# Postradical cystectomy bowel perforation caused by a drainage tube: a case report

**DOI:** 10.1186/1757-1626-1-350

**Published:** 2008-11-25

**Authors:** Eleftherios P Chatzidarellis, Andreas Skolarikos, Evangelos Mazaris, Iraklis Mitsogiannis, Gerasimos Alivizatos

**Affiliations:** 1 2nd Department of Urology Athens Medical School, Sismanoglio Hospital, Athens, Greece

## Abstract

**Introduction:**

Open drains are frequently placed in the abdominal cavity to prevent the collection of fluid or blood following major surgery.

**Case presentation:**

We describe a case of perforation of the large bowel caused by the drain tube placed in a 74-year-old patient who had undergone radical cystectomy for invasive bladder cancer.

## Introduction

Perforation of the large bowel secondary to pressure necrosis caused by open drainage tubes is an extremely rare complication following major intra-abdominal surgery. Currently only twelve cases been reported in the literature, all in general surgery operations [1-7]. We describe a similar case in a patient who underwent radical cystectomy for muscle-invasive bladder cancer. We also discuss possible predisposing and precipitating factors.

## Case presentation

A 74-year-old patient with a past medical history of diabetes, cerebral vascular disease and a moderate degree of cardiac insufficiency underwent radical cystectomy for an extensive T2G3N0M0 bladder tumor. Prior to surgery there was no evidence of preexisting bowel disease, while the patient had never been operated intraabdominally or undergone a preoperative colonoscopy. A left crossed renal ectopia was found, during the urologic evaluation which included an excretory urogram (IVU) and an abdominal computed tomography scan.

The patient was classified as ASA score III and his operation was considered of increased surgical difficulty because of the presence of the fused left renal ectopia abutting the bladder and iliac vessels. As a consequence a fully informed consent was signed preoperatively. The patient underwent an uneventful 4-hour radical cystectomy and an ileal conduit with bilateral end to side uretero-enteric anastomoses was created. The stoma was fashioned at the pre-selected site at the right lower abdominal quadrant.

**Figure 1:**
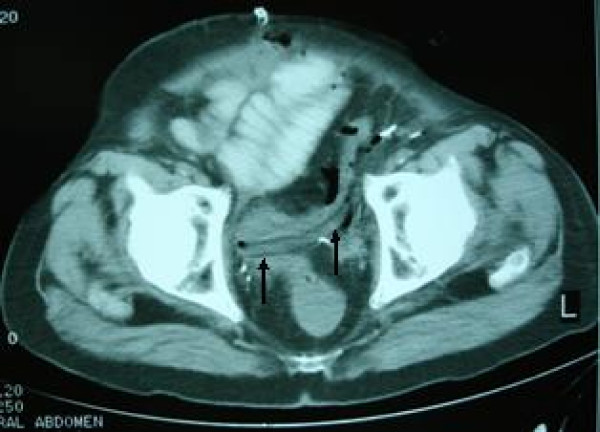
**CT scan at case presentation**. Computed tomography showing the drain abutting the sigmoid colon.

**Figure 2:**
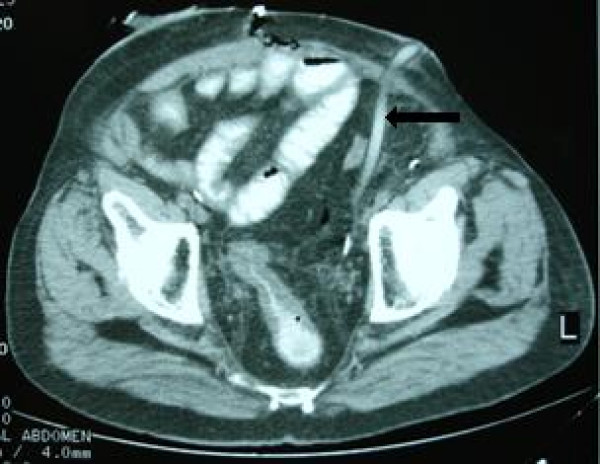
**CT scan at case presentation**. Computed tomography demonstrates gastrografin into the drain.

The peritoneal cavity was drained by an open-ended latex flexible tube of 8 mm in internal diameter which entered from the left lower quadrant and was placed at the pelvis, at a length of about 15 cm. The tube was draining to gravity, without any suction. The drain had also two side holes at its last 5 cm length, for better drainage. Patients' recovery was slow, as bowel mobilization was delayed but without clinical or radiographical confirmed ileus. As a consequence the drain remained in the aforementioned position, although its output did not exceed 200 ml for five days and there was no evidence of urine leakage. On the 6^th ^postoperative day, bowel content appeared in the drain. A contrast CT scan of the abdomen and pelvis revealed a patent and intact small bowel anastomosis but confirmed a sigmoid colon perforation. Recall of the surgical procedure and review of the CT films (Fig. 1, 2) indicated that the most likely cause of the perforation was "pressure necrosis" of the large bowel wall, secondary to the drainage tube apposition. The patient was treated conservatively with parenteral nutrition, intravenous antibiotics and repositioning of the tube away from the colon confirmed by a new CT scan. The initial decision for conservative management was based on the high mortality rate of re-operated patients described after radical cystectomy and due to absence of peritonitis. However, a laparotomy was performed on the 9^th ^postoperative day due to increased bowel content effluence and septicemia. The sigmoid colon perforation was confirmed, by finding a hole of about 5 cm in length and 1 cm in width and a Hartman's colectomy was performed. Although the drain had been withdrawn prior to the exploratory laparotomy, the shape of the bowel injury was consistent with an imprint of the drain, as shown in the CT scan preoperatively.(Fig 1) Patient's recovery was further complicated by an ischemic acalculous cholecystitis necessitating a cholecystectomy on postoperative day 14. On the 26^th ^postoperative day and due to ongoing and refractory sepsis the patient deceased. Histopathology of the resected left colon revealed ischaemic bowel disease with extensive bowel mucosa necrosis and atherosclerotic plaques and thrombotic lesions of the bowel wall vessels.

## Discussion

Bowel perforation caused by drainage tubes following abdominal surgery is a rare complication, with only twelve cases thus far reported in literature [1-7]. All cases occurred in general surgical procedures, while this rare complication has never been reported following Urological surgery. The underlying mechanism responsible for this complication differs depending on the type of the drains used. Bowel wall can be drawn into the side holes of a suction drain due to the creation of high negative pressure which can reach the level of -180 mmHg [8]. Open drains may cause perforation due to pressure necrosis by the tip of the tube. In addition, "stripping" of silicon surgical drains may increase the negative pressure to a level of -80 mmHg.[8] We retrospectively tried to identify predisposing or precipitating factors for this complication. Radical cystectomy in patients with fused pelvic "lump" kidneys has rarely been reported [9-11]. Three previous reports indicated a higher difficulty compared to formal cystectomies, mainly due to the alternate vascularization and to the shortness of the left ureter. However, in our case there was no difficulty in identifying the left ureter, while its length was adequate for re-implantation. The possibility of an iatrogenic injury of the bowel with the use of cautery, cannot be excluded but is considered highly unlikely, since ligation of vessels close to the bowel was only performed by sutures. Dissecting the sigmoid colon away was easy without any obvious serosal tears or injury of its arterial supply. Ischemic arterial disease was the main predisposing factor in our case as was indicated by the patient's medical history and according to the pathology report. The complication was precipitated by the late withdrawal of the drainage tube. This was mainly due to the delayed bowel function restoration in our patient. In order to avoid this complication, soft-type drains should be placed carefully without suction and removed or mobilized early after the drain fluid has decreased, especially when patients with vascular insufficiency are being operated on.

## Abbreviations

ASA: American Society of Anesthesiologists; CT: Computed Tomography.

## Consent

Written informed consent was obtained from the patient's sons for publication of this case report and any accompanying images. A copy of the written consent is available for review by the Editor-in-Chief of this journal.

## Competing interests

The authors declare that they have no competing interests.

## Authors' contributions

EPC, AS, EM, IM and GA conceived the study and participated in its design, coordination and helped to draft the manuscript. All authors read and approved the final manuscript.
